# Roll‐to‐Roll Manufacturing of Breathable Superhydrophobic Membranes

**DOI:** 10.1002/smtd.202400038

**Published:** 2024-04-09

**Authors:** Huan Liu, Haosong Zhong, Qiaoyaxiao Yuan, Rongliang Yang, Minseong Kim, Yee Him Timothy Chan, Siyu Chen, Jing Lin, Mitch Guijun Li

**Affiliations:** ^1^ Research Center on Smart Manufacturing Division of Integrative Systems and Design The Hong Kong University of Science and Technology Clear Water Bay, Kowloon Hong Kong SAR 999077 P. R. China; ^2^ State Key Laboratory of Advanced Displays and Optoelectronics Technologies The Hong Kong University of Science and Technology Clear Water Bay, Kowloon Hong Kong SAR 999077 P. R. China

**Keywords:** breathability, human perspiration, laser fabrication, roll‐to‐roll manufacturing, superhydrophobicity

## Abstract

Self‐cleaning and anti‐biofouling are both advantages for lotus‐leaf‐like superhydrophobic surfaces. Methods for creating superhydrophobicity, including chemical bonding low surface energy molecular fragments and constructing surface morphology with protrusions, micropores, and trapped micro airbags by traditional physical strategies, unfortunately, have encountered challenges. They often involve complex synthesis processes, stubborn chemical accumulation, brutal degradation, or infeasible calculation and imprecise modulation in fabricating hierarchical surface roughness. Here, a scalable method to prepare high‐quality, breathable superhydrophobic membranes is proposed by developing a successive roll‐to‐roll laser manufacturing technique, which offers advantages over conventional fabrication approaches in enabling automatically large‐scale production and ensuring cost‐effectiveness. Nanosecond laser writing and femtosecond laser drilling produce surface microstructures and micropore arrays, respectively, endowing the membrane with superior antiwater capability with hierarchical microstructures forming a barrier and blocking water infiltration. The membrane's breathability is carefully optimized by tailoring micropore arrays to allow for the adequate passage of water vapor while maintaining superhydrophobicity. These membranes combine the benefits of anti‐aqueous corrosive liquid behaviors, photothermal effects, thermoplastic properties, and stretchable performances as promising comprehensive materials in diverse scenes.

## Graphical abstract

High‐quality, breathable superhydrophobic membranes are developed by the two‐step successive roll‐to‐roll laser manufacturing technique for producing hierarchical surface microstructures and tailored micropore arrays, respectively. This study aims to overcome the challenges faced by chemical and traditional physical approaches in fabricating low surface energy and surface roughness for superhydrophobicity in an automatically scalable and cost‐effective way.

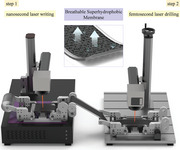

## 1 Introduction

Natural phenomena show superior water repellency. A water strider can walk on water freely with many dense microstructures on their legs.^[^ ^1^, ^2^, ^3^ ^]^ Likewise, lotus leaves are famous for their self‐cleaning performance with tiny papillae arrays and wax tubules grown on the surface.^[^ ^4^, ^5^, ^6^ ^]^ In Young's equation,^[^ ^7^, ^8^ ^]^ as shown in Equation (1),

(1) $$\;\cos\;\theta \;=\;\frac{\gamma_{\mathrm{SV}}\;-\;\gamma_{\mathrm{SL}}}{\gamma_{\mathrm{LV}}}$$

*γ* refers to surface tension, *S*, *V*, and *L* refer to solid, vapor, and liquid, respectively, and *θ* refers to the contact angle of a liquid droplet on the solid/liquid interface. According to Young's equation, when the liquid droplet reaches equilibrium on an ideal smooth solid surface in air, surface tensions and contact angle could be balanced; low surface energy substances could promote water repellency.^[^ ^9^, ^10^, ^11^, ^12^ ^]^ In reality, Cassie‐Baxter's theory is more appropriate since inevitable surface roughness should also be considered.^[^ ^13^, ^14^, ^15^ ^]^ Young's equation can be calibrated accordingly to Equation (2),

(2)

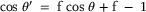




Theta *θ′* refers to the contact angle on a rough surface, *f* refers to the contact area fraction, and *f *< 1. In Cassie‐Baxter's theory, regulating surface energy by chemical bonding low surface tension molecular moieties, as well as constructing hierarchical surface morphology, plays pivotal roles in manipulating the wetting capability of a solid surface.^[^ ^5^, ^16^, ^17^, ^18^, ^19^, ^20^ ^]^ Therefore, we can get a theoretical explanation for the above miraculous behaviors. Low surface energy wax tubules on the outer layer can help repel dirt, moisture, water, etc., on lotus leaves. For another, the unique microstructures can reduce the contact area between water and solid surfaces,^[^ ^21^ ^]^ like the insect's legs and papillae arrays on lotus leaf surfaces, so that the water strider can walk on water and water droplets can roll off the leaves, flushing the contaminants away.

Many research ideas and strategies for fabricating superhydrophobicity have been devised based on Cassie–Baxter's theory and fruitful research work has been implemented worldwide in recent years. Zhang's group used solvent evaporation and fluorine/silicon modification to achieve surface roughness and low surface energy.^[^ ^22^, ^23^ ^]^ Ma's group prepared a porous aerogel structure by freeze‐drying polyacrylonitrile nanofibers, carbon nanotubes, and polyvinyl alcohol solution to build surface morphology.^[^ ^24^ ^]^ Similarly, Zu's group used the freeze‐drying method to synthesize a superhydrophobic triple network reduced graphene oxide aerogel in organosilane solution.^[^ ^25^ ^]^ Dong's group used 3D printing technology to construct a superhydrophobic interface using an ink composed of hydrophobic monomers. Nanoporous structures and bulk superhydrophobicity were produced by ink phase separation in photopolymerization.^[^ ^26^ ^]^ Superhydrophobicity‐related work has also been studied using laser technique. In Wang's work, surface wettability was compared by regulating the defocus level of laser writing.^[^ ^27^ ^]^ In Shao's work, laser writing and shape‐memory polymer pouring were combined to build a polymer replica with microscale structures. This was followed by the hydrophobic silica particle deposition to lower surface energy and increase surface roughness, endowing the surface with superhydrophobicity.^[^ ^28^ ^]^ However, introducing low surface energy molecular fragments through chemical bonding to develop superhydrophobic coatings can be complex and environmentally concerning. The process involves stubborn accumulation and brutal degradation, particularly for long fluorinated alkyl chains. These chains can harm the environment and creatures, and the disposal of chemical agents can also pose problems.^[^ ^29^, ^30^, ^31^, ^32^ ^]^ Conversely, superhydrophobic structures constructed by traditional physical approaches, including doping, solvent evaporation, freeze drying, phase separation, self‐assembling, etc., can effectively create hierarchical and heterogeneous micro/nanoscale surface morphology.^[^ ^33^, ^34^, ^35^, ^36^, ^37^ ^]^ However, the fabricated surfaces are usually not wear‐resistant and cannot suffer daily use.^[^ ^38^, ^39^ ^]^ The preparation process needs trials and exploration owing to the random size and distribution of protrusions, micropores, and trapped micro airbags resulting from infeasible calculation and imprecise modulation. Among these strategies, introducing low surface energy molecules containing Si moieties and exploiting the emerging laser technique show more appealing merits in human and environmental friendliness and ultra‐high efficiency.^[^ ^27^, ^40^, ^41^, ^42^, ^43^ ^]^ Superhydrophobic surfaces with stable reproducibility can be prepared in nanoseconds by finely regulating laser parameters.^[^ ^44^ ^]^

Herein, we report a breathable superhydrophobic (SHB) membrane prepared by the two‐step laser technique using a 304 stainless steel plate and a high‐performance parafilm composed of low surface energy waxes and polyolefins. The membrane precursor produced by the first‐step nanosecond laser writing showed overwhelming hydrophobicity with an average water static contact angle (SCA) more prominent than 150° and an average sliding angle (SA) smaller than 10°. Water droplets could easily roll off the membrane, leaving no residue. Subsequently, uniform micropore arrays were drilled by the femtosecond laser with tailored pore size and distribution, endowing the superhydrophobic membrane with breathability with an average relative humidity (RH) of ≈90% in the simulation experiment of human perspiration scenario. The membrane is uniformly black, giving it an excellent photothermal effect. After being irradiated under one sun by the solar simulator, it reached an average temperature of ≈75 °C. This makes it potentially useful as protective clothing and shoes for rainy days since it can help keep you dry and warm, providing enhanced protection and comfort. The parafilm‐based membrane exhibited resistance to corrosive aqueous liquids to a certain extent. It maintained remarkable lyophobicity even in a stretched state, making it suitable for various shapes and practical situations. Owing to its thermoplasticity, the membrane can be quickly melted under heat and adhered to a substrate surface like metal mesh. This allows for the creation of unique Janus membranes for further research and application purposes. Moreover, continuous roll‐to‐roll manufacturing fulfills automated processing, promoting productivity and enabling mass production for the membrane.

## 2 Results and Discussion

**Figure 1:**
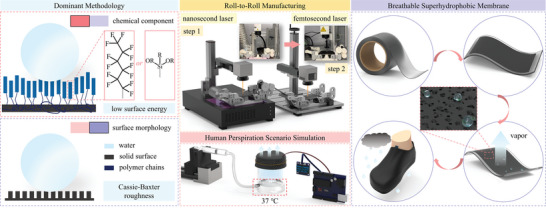
Illustration of the dominant methods for preparing superhydrophobic surfaces, roll‐to‐roll manufacturing of the breathable superhydrophobic membranes, a human perspiration scenario simulation experiment, and the relationship diagram of material/property/application of the prepared breathable superhydrophobic membranes.

An outstanding breathable superhydrophobic membrane lined with hierarchical surface roughness and microporous arrays exhibiting an average micropore size of 300 µm and pore distance of 1.41 mm was successfully prepared by a nanosecond pulse laser writing step and a subsequent femtosecond laser drilling step in continuous roll‐to‐roll manufacturing, as illustrated in **Figure** **1**. An Arduino‐based humidity sensor and the roll‐to‐roll configuration were devised, and a human perspiration scenario simulation experiment was implemented.

### 2.1 Morphological Structure and Chemical Composition Characterization

**Figure 2:**
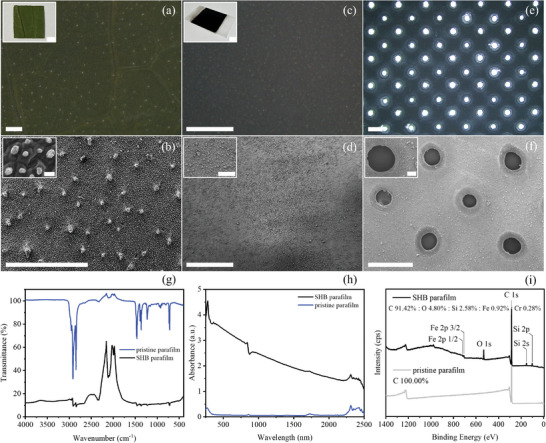
Morphological structure and chemical composition characterization of diverse surfaces. a) OM image and b) SEM image of the lotus leaf; the insets are the optical photo of the tested lotus leaf and the microstructure of the leaf surface with 2000X magnification, respectively; Scale bars from top to bottom are 10 mm, 1 mm, 10 µm, and 1 mm. c) OM image and d) SEM image of the superhydrophobic parafilm; the insets are the optical photo of the as‐prepared superhydrophobic parafilm and the surface roughness with 400X magnification, respectively; Scale bars from top to bottom are 10 mm, 1 mm, 100 µm, and 1 mm. e) OM image and f) SEM image of the as‐prepared breathable superhydrophobic parafilm; the average hole diameter is 300 µm and the hole distance is 1.41 mm; the inset is the hole with 200X magnification; Scale bars from top to bottom are 1 mm, 100 µm, and 1 mm. g) FTIR spectra, h) UV–vis spectra, and i) XPS spectra of the prepared superhydrophobic parafilm and the pristine parafilm.

Surface roughness was produced by the high‐energy nanosecond laser writing, with the intense laser beam penetrating through the parafilm, etching on the interface of the stainless steel plate, and exciting electrons to higher energy levels from the ground.^[^ ^45^, ^46^ ^]^ Micro/nanoparticles flow in high heat and motion composed of solid, liquid, gas, and plasma produced transiently will impact the interfaces and partially carbonize the waxes and polyolefins in the parafilm. After cooling down, the micro/nanoparticles were redistributed onto the interface, constructing the surface morphology with protrusions and the trapped airbags, forming a barrier layer and effectively making the solid surface resistant to water infiltration. OM test and SEM test in **Figures** **2**c,d, and S1c (Supporting Information) showed successful microstructure construction compared with the unique rough surface of lotus leaves (Figure 2a,b). Subsequently, uniform micropore arrays were drilled by the femtosecond laser with tailored pore size and distribution, endowing the superhydrophobic membrane with breathability (Figure 2e,f; Figures S1a,b,d, Supporting Information). The black hierarchical carbonized layer endowed a high absorption of intense laser energy and a beneficial piercing process on the membrane. Both the front and back sides show uniformity in the micropores array, and a flat membrane was obtained with qualified flexibility and robustness. Chemical composition identification conducted by FTIR spectra (Figure 2g), UV–vis spectra (Figure 2h), XPS spectra (Figure 2i; Figure S4b,c, Supporting Information), XRD spectra (Figure S4a, Supporting Information), and EDS mapping of the superhydrophobic membrane (Figure S2, Supporting Information) and the breathable superhydrophobic membrane (Figure S3, Supporting Information) further indicates surface morphology on the laser‐manufactured parafilm works as the dominant influencing factor for superhydrophobicity with less low surface energy chemical constituents detected.

### 2.2 Superhydrophobicity Investigation

**Figure 3:**
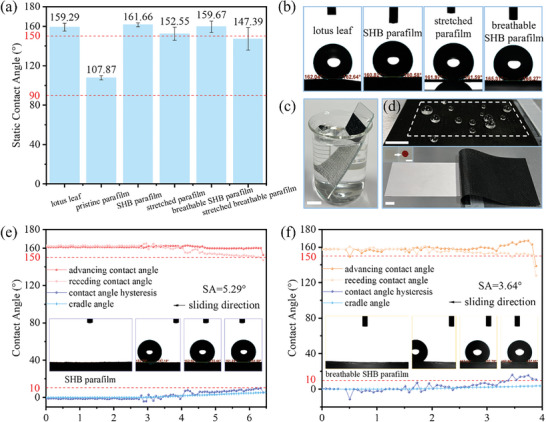
Water repellency capability investigation of different surfaces. a) the column graph of water static contact angles measured; b) the SCA images of lotus leaf, superhydrophobic parafilm, stretched hydrophobic parafilm, and the breathable superhydrophobic parafilm from left to right in sequence; Water droplets squeezed are ≈4 µL at room temperature. c) Silver mirror effect on the breathable superhydrophobic membrane; d) Antiwater performance of the breathable superhydrophobic membrane on discoloration test strips; The discoloration test strip was placed within the dashed box under the breathable superhydrophobic membrane with water droplets of 10–100 µL resting for 30 min. No color change was observed, indicating the non‐infiltration of water under the protection from the microstructure layer on breathable superhydrophobic membranes. The inset shows the test strip turning red when exposed to water. The average micropore diameter is 300 µm, and the pore distance is 1.41 mm. All the scale bars are 10 mm. Water dynamic contact angles were recorded at room temperature on e) the prepared superhydrophobic membrane precursor with a sliding angle of 5.29° in 6.48 s and f) the breathable superhydrophobic membrane with a sliding angle of 3.64° in 3.96 s. Water droplets squeezed are ≈4 µL.

Static and dynamic contact angles were thoroughly measured to examine the superhydrophobicity of the as‐prepared membranes using the contact angle meter with water droplets of 4 µL at room temperature. Static contact angles of 161.66°± 2.02° on the superhydrophobic parafilm and 159.67°± 5.75° on the breathable superhydrophobic parafilm were achieved compared with the control groups of lotus leaves with static contact angles of 159.29°± 3.93° and pristine parafilm with contact angles of 107.87°± 2.12°. Even when stretched twice the length, the membranes also possessed competitive hydrophobic capabilities with contact angles of 152.55°± 6.59° on the hydrophobic parafilm and 147.39°± 11.35° on the breathable hydrophobic parafilm (**Figure** **3**a,b). Outstanding antiwater repellency enables water droplets of ≈4 µL to slide down with a sliding angle of 5.29° in 6.48 s on the superhydrophobic parafilm and a sliding angle of 3.64° in 3.96 s on the breathable superhydrophobic parafilm (Figure 3e,f; Video S1, Supporting Information). Droplets were pinned firmly on the pristine parafilm surface even when it was turned over for 180°. The average sliding angles detected were 4.33°± 2.39° on the superhydrophobic parafilm and 5.81°± 2.46° on the breathable superhydrophobic parafilm with the counterpart stretched hydrophobic parafilm showing a sliding angle of 8.14°± 5.84° and the stretched breathable hydrophobic parafilm showing 16.12°± 4.41°, as listed in **Table** **1**. Meanwhile, outstanding superhydrophobicity is usually accompanied by a magical silver mirror effect.^[^ ^36^, ^47^, ^48^, ^49^, ^50^, ^51^ ^]^ A thin silver layer could be observed when the superhydrophobic membrane was submerged under water (Figure 3c), demonstrating the Cassie–Baxter roughness on the membrane surface and visualizing the fabricated micropore arrays.^[^ ^52^ ^]^ The breathable superhydrophobic membrane's antiwater performance was tested by placing a discoloration test strip under the membrane and adding water droplets of varying sizes for 30 min. The surface microstructures effectively protected the test strip from water infiltration without showing color change (Figure 3d). The durability and reliability of superhydrophobic membranes were investigated against water impact, tension, and mechanical abrasion, and satisfactory experimental results were obtained, as shown in Figures S6,S7,S9a, Video S4,S5, and S6 (Supporting Information).

**Table 1:** Water sliding angles were measured on different surfaces.

Surface	Pristine	SHB	Stretched	Breathable SHB	Stretched breathable
SA (°)	pinned	4.33°± 2.39°	8.14°± 5.84°	5.81°± 2.46°	16.12°± 4.41°

### 2.3 Breathability Evaluation

**Figure 4:**
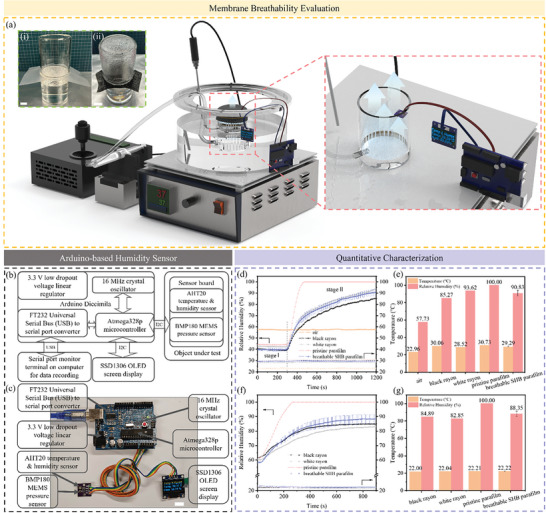
Membrane breathability investigation. a) Illustration of the human sweat scenario simulation experimental configuration and membrane breathability test; Insets show the breathability comparison between i) the pristine parafilm and ii) the breathable superhydrophobic membrane over water placed on the 100 °C heating plate for 30 min. Scale bars are 10 mm. b) the layout and working principle of the self‐devised Arduino‐based humidity and temperature sensor; c) the profile display comprised of individual components; Scale bar is 10 mm. d) Real‐time RH and temperature changes of black rayon, white rayon, pristine parafilm, and the breathable superhydrophobic membrane in the 37 °C oil bath pot, and the ambient air; stage I refers to the period during which the system reached equilibrium in heat and humidity; stage II refers to the vapor augmentation process in human perspiration scenario simulation. e) Breathability comparison among diverse materials at 37 °C; f) Real‐time RH and temperature changes of black rayon, white rayon, pristine parafilm, and the breathable superhydrophobic membrane at room temperature; g) Breathability comparison among diverse materials at room temperature.

To examine the breathability of the membranes, vapor condensation phenomena were compared between pristine parafilm and the breathable superhydrophobic membrane over water placed on the 100 °C heating plate for 30 min. As shown in **Figure** **4**‐i,ii, a water droplet layer formed on the bottom surface of the pristine parafilm while mist covered the entire inner wall of the cell above the prepared membrane, demonstrating excellent vapor permeability. A human perspiration scenario simulation experiment was thoughtfully devised to investigate the membrane breathability quantitatively, as illustrated in Figure 4a. Based on the body surface area (BSA) of 1.6 m^2^,^[^ ^53^, ^54^, ^55^ ^]^ one‐foot surface area (FSA) of BSA×3.5%,^[^ ^56^, ^57^ ^]^ and the sweat rate of 0.2–1 µL cm^−2^ min^−1^ in healthy individuals,^[^ ^58^ ^]^ a microfluidic configuration was designed and exploited. An Arduino‐based humidity sensor was developed (Figure 4b,c) to monitor and record relative humidity and temperature changes throughout the experiment. Afterward, the experimental setup included a motorized positioning system, a microfluidic system, a beaker wrapped with the breathable superhydrophobic membrane, and the sensor was assembled, as shown in Figure 4a and Figure S5b (Supporting Information). The syringe, the extrusion device, was positioned on the motorized platform, enabling precise control over the fluid extrusion rate by regulating the motor's movements (Figure S5i, Supporting Information). Breathability was quantitively evaluated and compared among different materials, such as black rayon, white rayon, pristine parafilm, and the prepared breathable superhydrophobic membrane (Figure S5a,c, and Video S2, Supporting Information). In the first 5 min, the experimental system reached an equilibrium in humidity and heat transfer. As the experiment unfolded, the fluid began infiltrating into the beaker. Water vapor concentration experienced a noticeable augmentation correlating with heightened evaporating rates, seeking an outlet for dissipation. The sole avenue available is the upper opening of the beaker, which was entirely covered by the prepared breathable superhydrophobic membrane. With the Arduino‐based humidity sensor diligently recording the fluctuating humidity levels, this orchestrated interplay between fluid introduction and the impediment of vapor escape through the porous membrane enables us to investigate RH levels for the accuracy and robustness of our research findings. During the 15‐min investigation in the ambient air with a humidity of 57.73% and a temperature of 22.96 °C, RH in the beaker covered by the breathable superhydrophobic membrane rose to 90.83% ± 3.25% with the counterpart black rayon of 85.27%, white rayon of 93.62%, and pristine parafilm of 100% when the whole setup was heated to 37 °C, as shown in Figure 4d,e. An RH of 88.35% ± 3.01% was achieved at room temperature, with the counterpart black rayon of 84.89%, white rayon of 82.85%, and pristine parafilm of 100% (Figure 4f,g), proving qualified breathability when used as protective clothing.

### 2.4 Intrinsic Qualities Exploration

**Figure 5:**
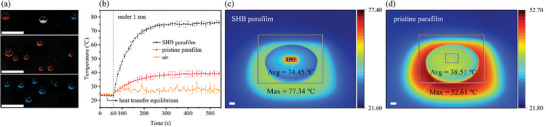
The intrinsic characteristic investigation of the prepared uniformly black superhydrophobic parafilm. a) the comprehensive anti‐aqueous corrosive liquids performance of the superhydrophobic parafilm against strong acid of 1 m HCl aqueous solution (pH 0), strong alkaline of 1 M KOH aqueous solution (pH 14), and deionized water (DI water) (pH 7) during a 10‐min evaluation; Scale bars are 10 mm. b) Photothermal effect study in temperature changes with time during 550‐s irradiation under one sun detected by the thermocouple device with the highest temperature of 76.63 ± 1.66 °C on the superhydrophobic parafilm, 39.53 ± 2.14 °C on the pristine parafilm, and 30.23 ± 1.45 °C in air; the corresponding thermal images captured by an infrared camera with c) an average temperature of 74.45 °C and a maximum temperature of 77.34 °C on the superhydrophobic parafilm and d) an average temperature of 38.51 °C and a maximum temperature of 52.61 °C on the pristine parafilm; Scale bars are 10 mm.

Superhydrophobicity is not limited to pure water. The membrane was discovered to possess a satisfactory anti‐aqueous corrosive liquid performance against strong acids of pH 0 and strong alkaline with pH 14 in the 10‐min test, as shown in **Figure** **5**a. Additionally, the anticorrosive capability was investigated more comprehensively at long‐time exposure and in a stretched state (Figures S8 and S9b, and Video S7, Supporting Information). The photothermal effect was also studied due to the uniformly black color on the prepared superhydrophobic membrane. During a 550‐s test, the highest temperature of 76.63 ± 1.66 °C on the prepared superhydrophobic membrane was recorded with the counterpart pristine parafilm of 39.53 ± 2.14 °C and air of 30.23 ± 1.45 °C, as shown in Figure 5b–d.

### 2.5 Roll‐to‐Roll Manufacturing Development

At last, the superhydrophobic parafilm and the breathable superhydrophobic parafilm were produced automatically by employing the self‐developed roll‐to‐roll machine with two different power systems distributed to nanosecond laser writing and femtosecond laser drilling processes, respectively. Continuous material conveyance, direct laser processing, and automatic membrane production can be achieved under the harmonious working mode between the laser machine and the roll‐to‐roll machine. By a two‐phase stepper motor electrically connected to the reserved rotational axis port and operated via the laser controlling software, the roll‐to‐roll machine worked in conjunction with the nanosecond laser writing machine equipped with a rotary segmentation marking function in Step 1 to prepare the superhydrophobic membrane (Video S3a, Supporting Information). By incorporating an Arduino‐based external power component in the roll‐to‐roll femtosecond laser drilling process in Step 2, precursor membranes obtained from Step 1 could be transported for successive micropore arrays drilling, enabling incessant fabrication and simultaneous collection of the breathable superhydrophobic membrane (Video S3b, Supporting Information).

## 3 Conclusion

To summarize, an outstanding breathable superhydrophobic membrane possessing comprehensive properties of excellent photothermal effect, thermoplasticity, and stretchability was prepared using the continuous roll‐to‐roll laser manufacturing technique in an ultra‐high efficient, cost‐effective, and environment‐friendly way. The superior water repellency protected the membrane from being wetted by aqueous liquids, and the uniformly distributed micropore arrays endowed it with high quality and competitive breathability. An Arduino‐based humidity sensor was developed to investigate the membrane's breathability in precisely controlled simulating situations for human sweating. A roll‐to‐roll laser manufacturing machine powered by two sources was also devised and fulfilled. This machine has been instrumental in enabling automated, successive fabrication and scalable mass production of breathable superhydrophobic membranes. These membranes are friendly on rainy days by helping dry clothing and shoes and keeping the wearer warm and comfortable.

## 4 Experimental Section

### Materials and Instruments

Parafilm of Bemis PM‐999, hydrochloric acid (HCl) of 36.5 wt.%, potassium hydroxide (KOH), sodium chloride (NaCl), microscope slides were purchased from Center of Laboratory Supplies in HKUST; 304 stainless steel plates were purchased from Guangdong Hongwang New Material Technology Co., Ltd; dyes were purchased from Henan Tanjiao Education Technology Co., Ltd; discoloration test strips for water were purchased from Shenzhen Chuangbaoda Technology Co., Ltd; the adhesive tape was purchased from Minnesota Mining and Manufacturing; the 50 mL electrolytic cells were purchased from Shanghai Jingchong Electronic Technology Development Co., Ltd; the JF946‐2020 heating table was purchased from Xiamen Weitek Electronics Co., Ltd; the beaker was purchased from Breeze glass shop; the latex tube and the syringe were purchased from Anhui Chixin Biotechnology Co., Ltd; the silicone glue of Kafuter K‐705 was purchased from Dongguan Kaida Technology Industry Development Co., Ltd; the black rayon and the white rayon were purchased from Cotton and Chemical Fiber Co., Ltd; the cast aluminum rollers and fittings with self‐devised structure were manufactured by Dongguan Huaying Machinery Equipment Co., Ltd; the polylactic acid (PLA) and the thermoplastic polyurethane (TPU) filament for 3D printing were purchased from Tuozhu Technology Co., Ltd; the 1064 nm pulse laser machine was purchased from Guangdong Dazu Yue Ming Laser Group Co., Ltd; the 1030 nm femtosecond laser machine was purchased from Wuhan Yangtze Soton Laser Co., Ltd; and the optical power meter for calculating laser energy and light intensity was obtained from FieldBest.

### Fabrication of the Breathable Superhydrophobic Parafilm

A high‐performance parafilm with flexible, moisture‐resistant, and self‐sealing advantages commonly used in laboratories and homes and a 304 stainless steel plate were employed to prepare the superhydrophobic membrane by laser writing using a 1064 nm pulse laser machine with the operating parameters of 250 mm s^−1^ in scanning speed, 15 W in working power, 20 kHz in laser frequency, 110 ns in pulse width, and 25 µm in filling interval. The laser energy and light intensity on every pulse were calculated at 70.28 J cm^−2^ and 6.39 × 10^8^ J cm^−2^ s^−1^, respectively, according to Equations (3) and (4). The breathable superhydrophobic parafilm was subsequently prepared by drilling micropores using a 1030 nm femtosecond laser machine with the operating parameters of 1 mm s^−1^ in drilling speed, 600 W in working power, 5 MHz in laser frequency, 3000 in step value, and 300 fs in pulse width. The laser energy and light intensity on every pulse were calculated at 0.12 J cm^−2^ and 4 × 10^10^ J cm^−2^ s^−1^, respectively.

(3) $$E\;=\;4P\;/\;\pi \;F\;\phi^{2}$$

(4) $$I\;=\;E\;/\;\tau \;\;=\;4P\;/\;\pi \;\tau \;F\;\phi^{2}$$

In which *E* refers to laser energy, *P* refers to the power detected by the optical power meter, *F* refers to the pulse laser frequency, *ϕ* refers to the diameter of the laser spot, *I* refers to light intensity, and *τ* refers to pulse width.

### Characterization

The morphological structure was characterized using a Hayear optical microscope (OM) and a JEOL‐7100F scanning electron microscopy (SEM). The chemical composition was analyzed using an Oxford energy dispersive spectrometer (EDS), a Vertex 70 Fourier transform infrared (FTIR) spectrometer, a PerkinElmer UV–vis spectrometer, a Kratos Axis Ultra DLD X‐ray photoelectron spectroscopy (XPS) analysis system, and a PANalytical X‐ray diffractometer (XRD). The antiwater performance was studied using a Biolin Theta contact angle (CA) meter. The photothermal effect was investigated using a Newport solar simulator, a UT321 thermometer, and a Fluke Ti480 Pro infrared (IR) thermal imager. The breathability test used a PDV PP110‐30‐5040 high‐precision motorized linear stage and a KZ‐100 motion controller.

### Fabrication and Working Mechanism of the Humidity Sensor

The humidity sensing device was made with an AHT20 humidity sensor, an Arduino UNO readout unit, and an SSD1306 organic light‐emitting diode (OLED) display module. The AHT20 humidity sensor and SSD1306 OLED display screen were connected to the microprocessor (Atmega328P) on the Arduino board via the inter‐integrated circuits (IIC) bus. The program was written and compiled in the Arduino integrated development environment (IDE) using peripheral libraries from Adafruit (Adafruit_SSD1306 and Adafruit_AHTX0). When the Arduino UNO powers up, it initializes the serial port, display, and sensor via the IIC bus and enters the primary looping function. In the looping function, the Arduino requests data from the AHT20 sensor, which is displayed on the OLED screen and traced via the serial port.

### Breathability Evaluation

The experimental setup was thoughtfully designed and encompassed various components, including a microfluidic system, a motorized positioning system, an Arduino‐based humidity sensor, and the target laser‐prepared superhydrophobic porous parafilm. The liquid extrusion system, including a 10 mL syringe, a 200 mL beaker equipped with one single inlet port, and a latex tube with a narrow channel diameter of 0.3 mm, was assembled and positioned on a motorized platform for simulating human sweating situations. To ensure a watertight connection, the linkages of the pipe between the syringe and the bottom inlet of the beaker were securely sealed with silicone glue. The sensor was placed inside the top of the beaker, wrapped by the breathable membrane, recording humidity changes throughout the experiment. The rate of fluid extrusion could be calculated according to Equations (5) and (**6**), and achieved by precise control over the motor's movements.

(5) V=Arτ

(6) v=L/τ

Here, *V* refers to the volume of the sweat a healthy human produces during a 1000‐s test, *A* refers to one‐foot surface area (FSA), *r* refers to human sweat rate, *τ* refers to the test duration, *ν* refers to the fluid extrusion rate, and *L* refers to the length on the 10 mL syringe at which the volume of the liquid inside is equal to *V*. Based on 1 µL cm^−2^ min^−1^ as the sweat rate, the experiment commenced with a continuous flow of 1 wt.% NaCl solution at a consistent rate of 0.054 mm s^−1^ in the 10 mL syringe, simulating the volume and speed of human sweat in an oil bath pot at 37 °C for 15 min. Water vapor escaped incessantly through the micropores of the breathable membrane with vapor concentration augmentation. The real‐time RH levels and temperature were shown on the sensor screen, and data was simultaneously transmitted to a computer for meticulous documentation and analysis (Figure S5b, Supporting Information).

### Fabrication and Working Mechanism of the Roll‐to‐Roll Machine

The roll‐to‐roll machine, which employs a configuration comprising two stationary aluminum‐based rollers for membrane conveyance, three adjustable PLA rollers for materials output and collection, a mechanical supporting bracket for device integration, gear sets, and two pivotal power systems was devised and constructed for the automatically successive production of the breathable superhydrophobic membrane. The rollers were 35 mm in diameter and 120 mm in length. There were two categories of gears. The driving gear had a module of 1.5, a tooth count of 43, and a maximum diameter of 67.5 mm. The two interconnected gears, equipped with the rollers, had a module of 1.5, a tooth count of 34, and a maximum diameter of 54 mm. The power plant had a gear ratio of 34:43. The overall structure of the roll‐to‐roll configuration was fabricated with aluminum castings and 3D‐printed PLA modules. Two power systems were distributed to the nanosecond laser writing and femtosecond laser drilling processes, respectively. The powered motor continuously propelled the raw material and precursor membrane by inducing rotation between two collecting rollers at the end. Each roller has two half‐cylinders that securely clamp the membrane in the commissure for subsequent rolling collection. With the materials passing through the device, the pair of collection rollers rolled up the laser‐processed membranes with the spare paper cover collected by the lower roller for resource recycling and environment protection purposes.

In Step 1, the roll‐to‐roll machine worked with the nanosecond laser writing machine with a rotary segmentation marking function. The NEMA17 two‐phase stepper motor was electrically connected to the reserved rotational axis port of the laser machine and operated via the laser controlling software. The segmentation distance was 0.5 mm, the pulse number of each revolution was 500, the filling interval was 0.05 mm, the scanning speed was 250 mm s^−1^, the laser frequency was 20 kHz, and the working power was 15 W. When laser writing was started and enabled with continuous marking mode, the motor would roll the laser‐processed membrane by 0.5 mm once the width of the laser marked area reached 0.5 mm. The galvo would repeat the scanning pattern and path for the incessantly automated fabrication of the superhydrophobic membrane.

In Step 2, the roll‐to‐roll femtosecond laser drilling process was implemented by equipping it with an external power component. The smooth operation was controlled between the roll‐to‐roll machine and the laser machine by using the stepper library and creating a logic for running. The proprietary code system was stored in the Arduino UNO development board, which was linked to the stepper motor driver, providing power to the 42‐step motor and allowing it to move around. To achieve continuous roll‐to‐roll femtosecond laser drilling, the precursor membrane was programmed to progress 80 mm at a rate of 5 mm s^−1^ for a cyclical duration of 150 s. By employing a pair of identical rollers, the covering membrane could be clung closely onto the surface of the stainless steel plate, allowing for the successful laser scanning process, continuous membrane transportation, and simultaneous product collection workflow.

## Conflict of Interest

The authors declare no conflict of interest.

## Author Contributions

H.L., R.Y., and M.G.L. worked on the methodology devising and experimental implementation. H.Z. and Q.Y. worked on software and configuration development. M.K. worked on illustration and visualization. Y.H.T.C., S.C., and J.L. assisted in sample testing. H.L. processed data and drafted the manuscript. M.G.L. guided and supervised the overall work. All authors reviewed and finalized the manuscript.

## Supporting information

Supporting Information

Supplemental Videos 1–4

Supplemental Videos 5–7

## Data Availability

The data that support the findings of this study are available from the corresponding author upon reasonable request.
