# Supplementary material for: Roll‐to‐Roll Manufacturing of Breathable Superhydrophobic Membranes
Source: Small Methods. 2024 Apr 9;8(12):2400038. doi: 10.1002/smtd.202400038 (PMC11672173; doi:10.1002/smtd.202400038)
Supplement: Supplementary file 1 — Supporting Information [file SMTD-8-2400038-s002.docx]

Supporting Information

**Roll-to-Roll Manufacturing of Breathable Superhydrophobic Membranes**

*Huan Liu, Haosong Zhong, Qiaoyaxiao Yuan, Rongliang Yang, Minseong Kim, Yee Him Timothy Chan, Siyu Chen, Jing Lin, Mitch Guijun Li**

List of contents

**Section 1.** Morphological structure characterization of the as-prepared superhydrophobic parafilm and the breathable superhydrophobic membrane.

**Figure S1.** OM and SEM characterization of the prepared superhydrophobic parafilm and the breathable superhydrophobic membrane.

**Section 2.** Chemical composition characterization of the superhydrophobic parafilm and the breathable superhydrophobic membrane.

**Figure S2.** EDS mapping and elemental analysis of the superhydrophobic parafilm with (a) 50X magnification and (b) 100X magnification.

**Figure S3.** EDS mapping and elemental analysis of the breathable superhydrophobic membrane with (a) 30X magnification and (b) 100X magnification.

**Figure S4.** (a) XRD spectra and (b-c) XPS spectra of the superhydrophobic parafilm and the pristine parafilm.

**Section 3.** Breathability Evaluation.

**Figure S5.** Breathability evaluation of diverse materials.

**Section 4.** Durability Investigation.

**Figure S6.** Water impact resistance test of the prepared superhydrophobic membrane.

**Figure S7.** Abrasion resistance test.

**Figure S8.** Anti-corrosive liquids capability investigation.

**Figure S9.** Liquid-repellent capability investigation on superhydrophobic membranes in the stretched state of 5 times in length.

**Section 5.** The anticipated application of the breathable superhydrophobic membrane serving for protective shoes.

**Figure S10.** The prototype of the shoes.

**Section 6.** Videos.

**Video S1.** Sliding angle test at room temperature. (a) the sliding process of a 3.95 μL water droplet on the superhydrophobic parafilm with a sliding angle of 5.29°; (b) the sliding process of a 3.78 μL water droplet on the breathable superhydrophobic membrane with a sliding angle of 3.64°.

**Video S2.** Breathability evaluation experiment of the breathable superhydrophobic membrane with an average pore diameter of 300 μm and a pore distance of 1.41 mm in the 37 ℃ oil bath pot for 16 mins. Data points were collected every ten seconds; Video S2 was played with a 100X fast-forwarding speed.

**Video S3.** Roll-to-roll manufacturing process. (a) the first step in preparing the superhydrophobic parafilm with a 500X fast-forwarding playing speed and (b) the second step in preparing the breathable superhydrophobic membrane with a 50X fast-forwarding playing speed in drilling and a 10X fast-forwarding playing speed in rolling.

**Video S4.** Anti-water demo of the as-prepared breathable superhydrophobic membrane for anticipated shoe application with a 3X fast-forwarding playing speed.

**Video S5.** Water impact resistance test.

**Video S6.** Abrasion resistance test.

**Video S7.** Anti-corrosive liquids capability investigation.

**Section 1.** Morphological structure characterization of the as-prepared superhydrophobic parafilm and the breathable superhydrophobic membrane.


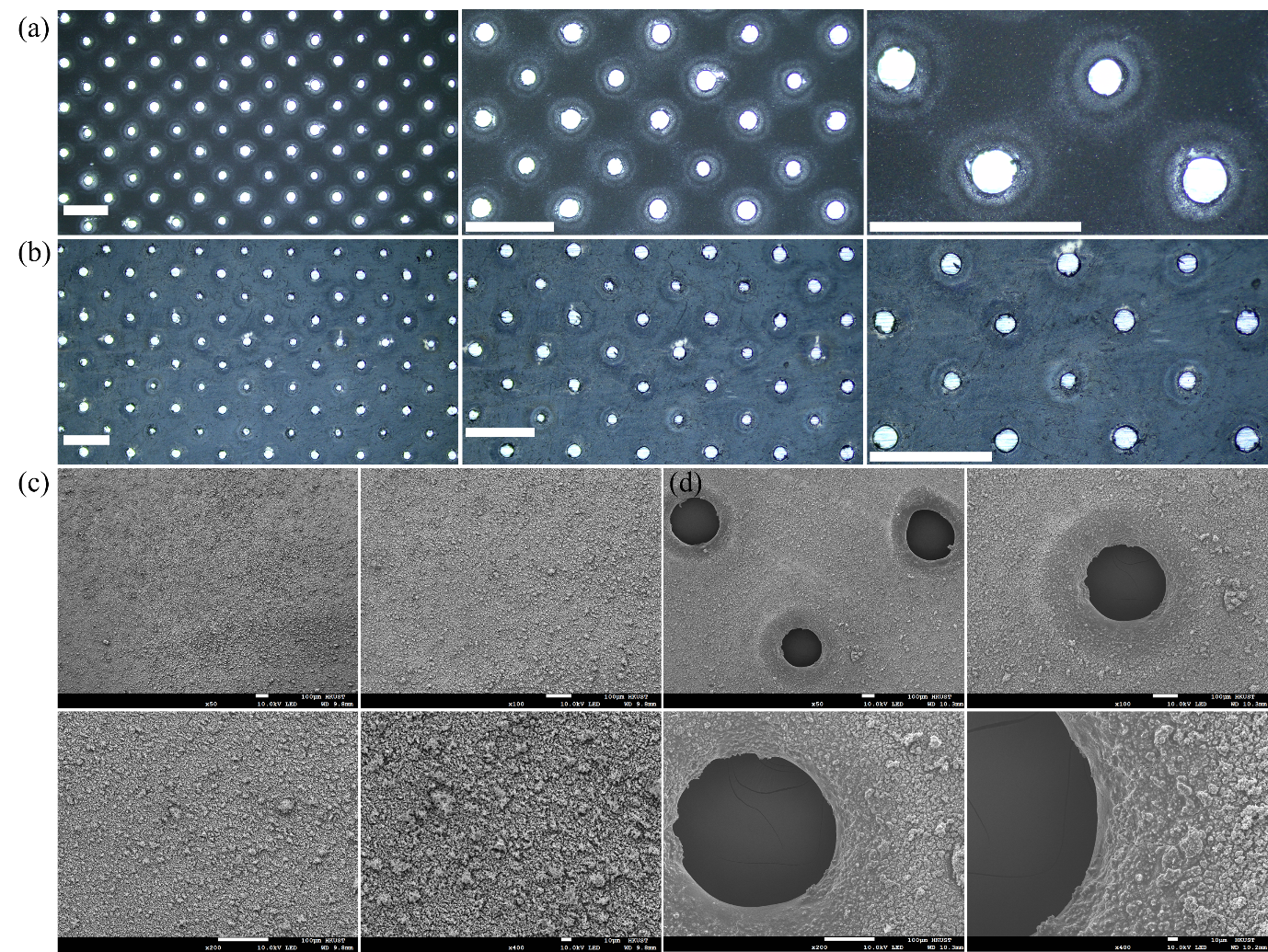
**Figure S1.** OM and SEM characterization of the prepared superhydrophobic parafilm and the breathable superhydrophobic membrane. OM images of the breathable superhydrophobic membrane on (a) the front side and (b) the back side with different magnifications. The average pore diameter is 300 μm and the pore distance is 1.41 mm. All the scale bars in Figures S1a and S1b are 2 mm. SEM images of (c) the superhydrophobic parafilm with different magnifications and (d) the breathable superhydrophobic membrane with different magnifications.

**Section 2.** Chemical composition characterization of the superhydrophobic parafilm and the breathable superhydrophobic membrane.


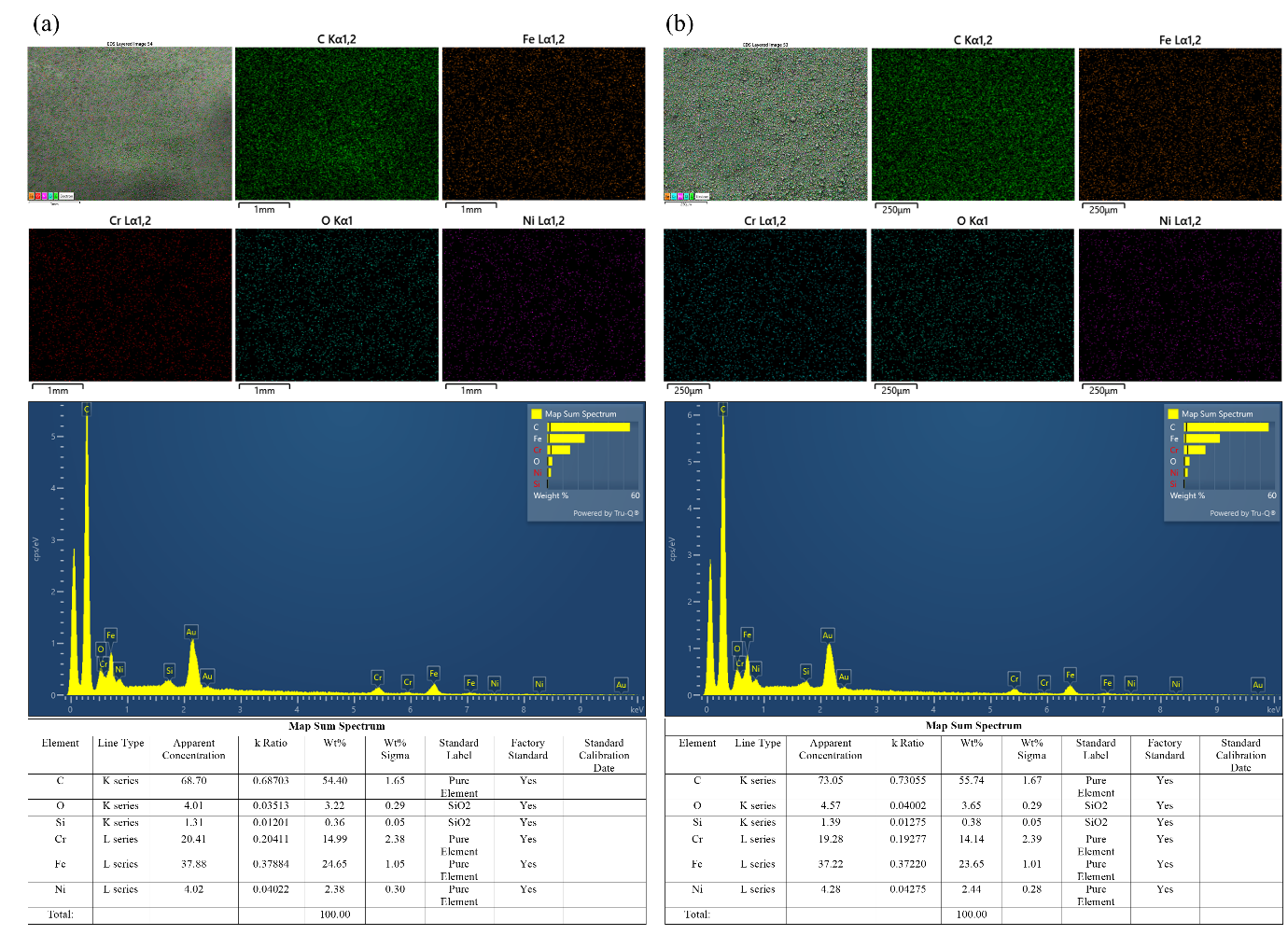
**Fig****ure S2.** EDS mapping and elemental analysis of the superhydrophobic parafilm with (a) 30X magnification and (b) 100X magnification.


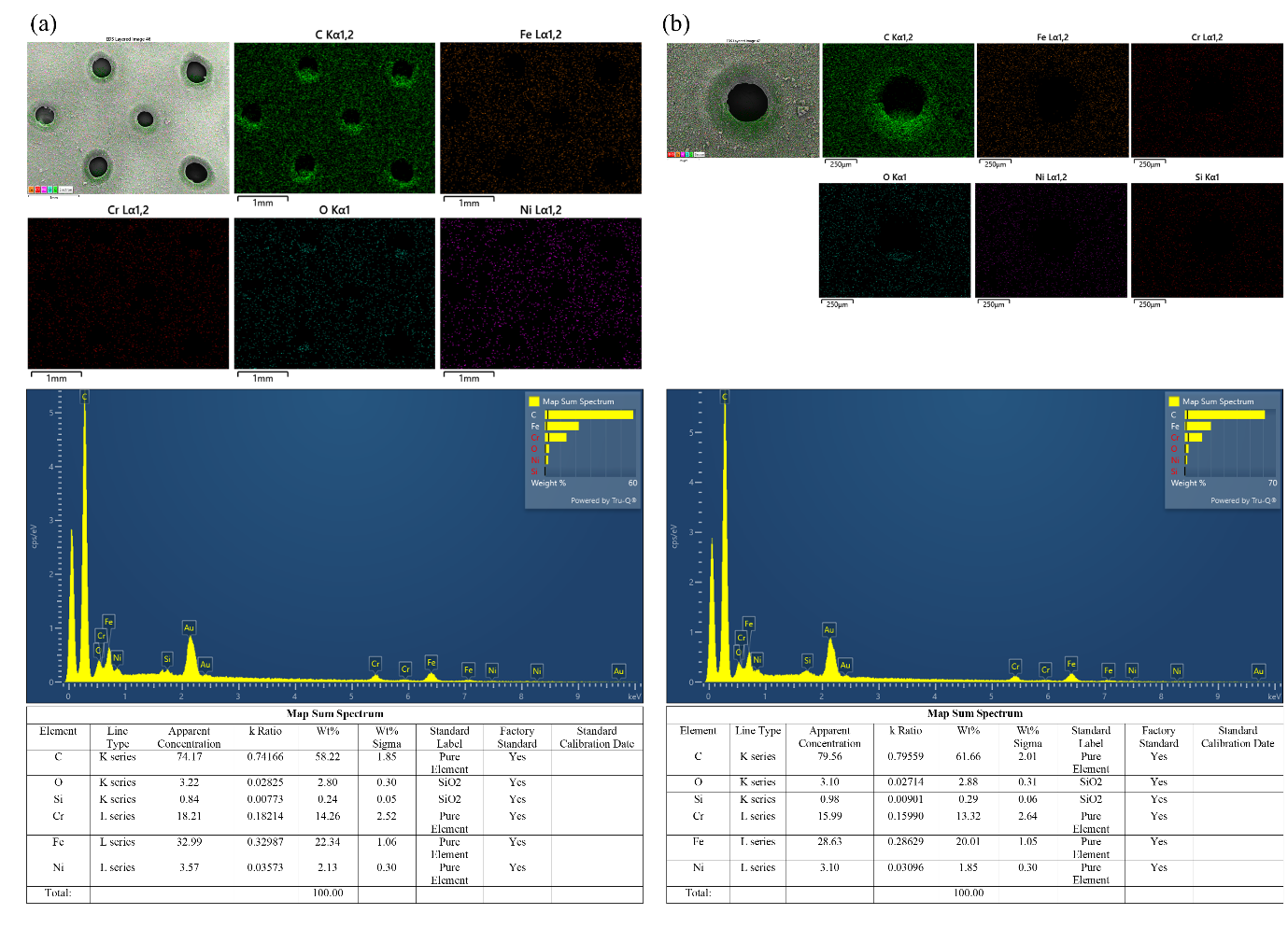
**Figure S3.** EDS mapping and elemental analysis of the breathable superhydrophobic membrane with (a) 30X magnification and (b) 100X magnification.


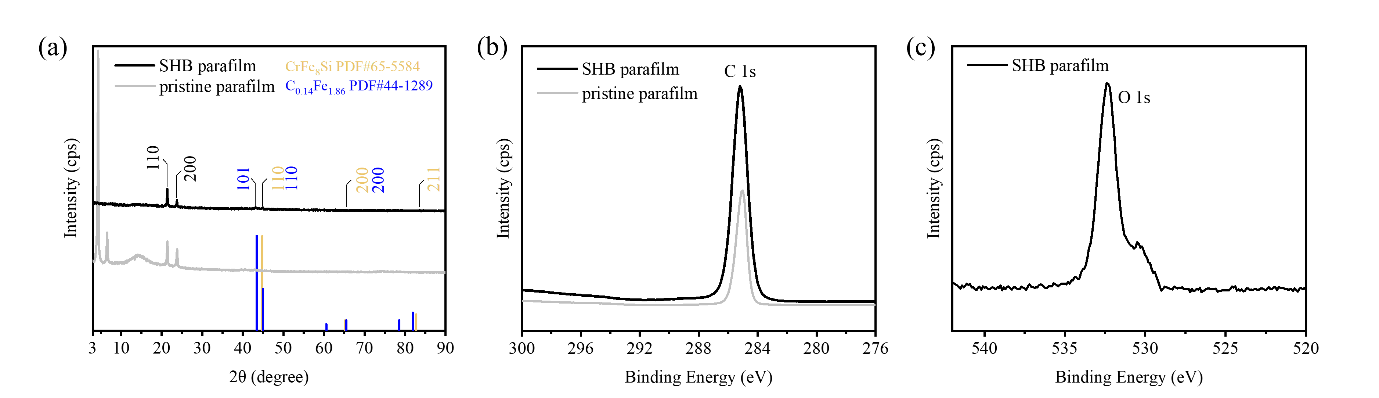
**Figure S4.** (a) XRD spectra and (b-c) XPS spectra of the superhydrophobic parafilm and the pristine parafilm.

**Section 3.** Breathability Evaluation.


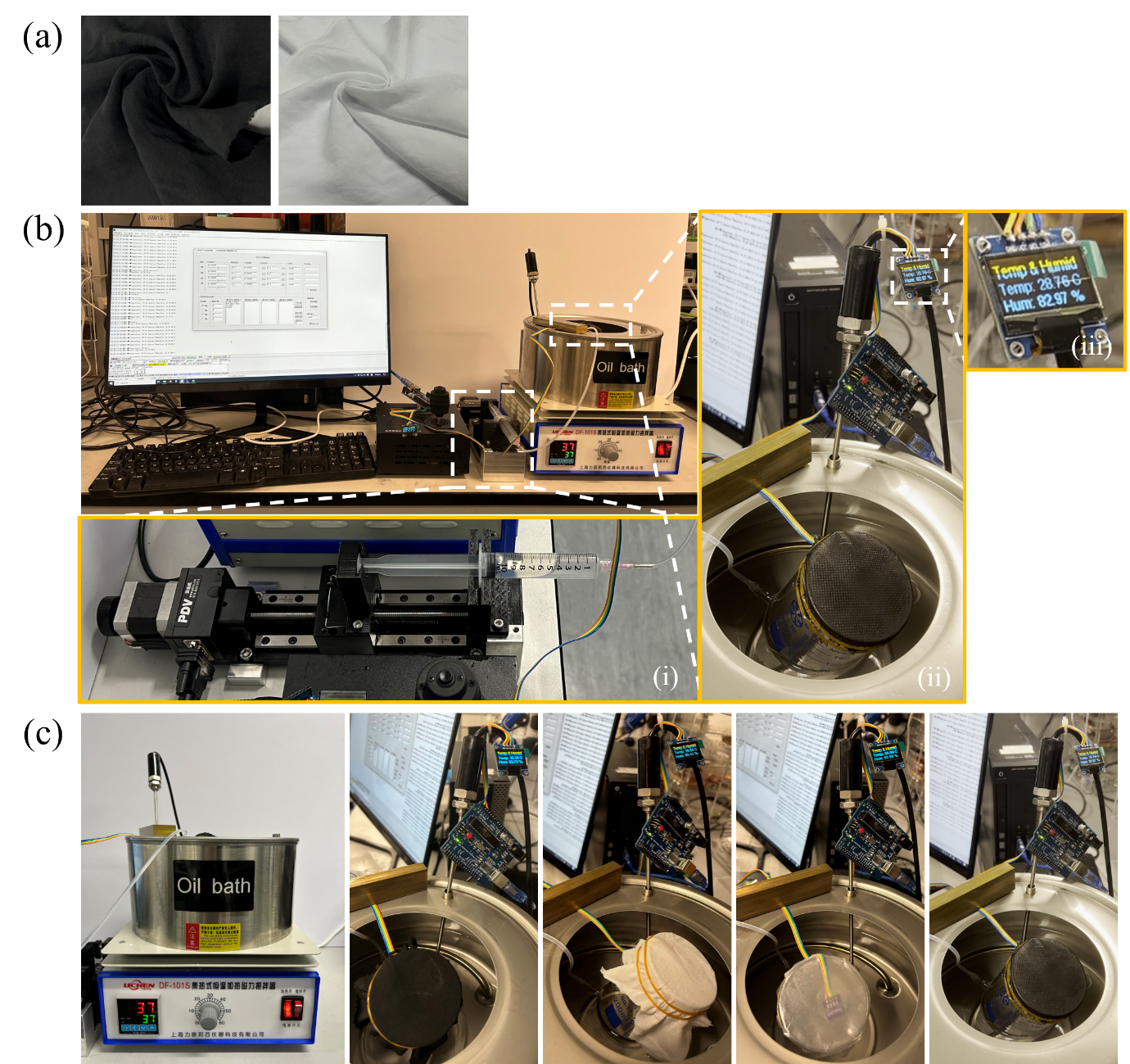
**Figure S5.** Breathability evaluation of diverse materials. (a) Black rayon and white rayon; (b) the whole setup of the breathability investigation experiment to simulate human sweat amount and rate in the 37 ℃ oil bath pot by operating the motorized positioning system; (i) the liquid extrusion system; (ii) the breathable superhydrophobic membrane against water vapor; (iii) the self-developed Arduino-based sensor recording the real-time humidity and temperature; (c) Breathability evaluation and comparison among different materials; from left to right: black rayon, white rayon, pristine parafilm, and the prepared breathable superhydrophobic membrane.

**Section 4.** Durability Investigation.


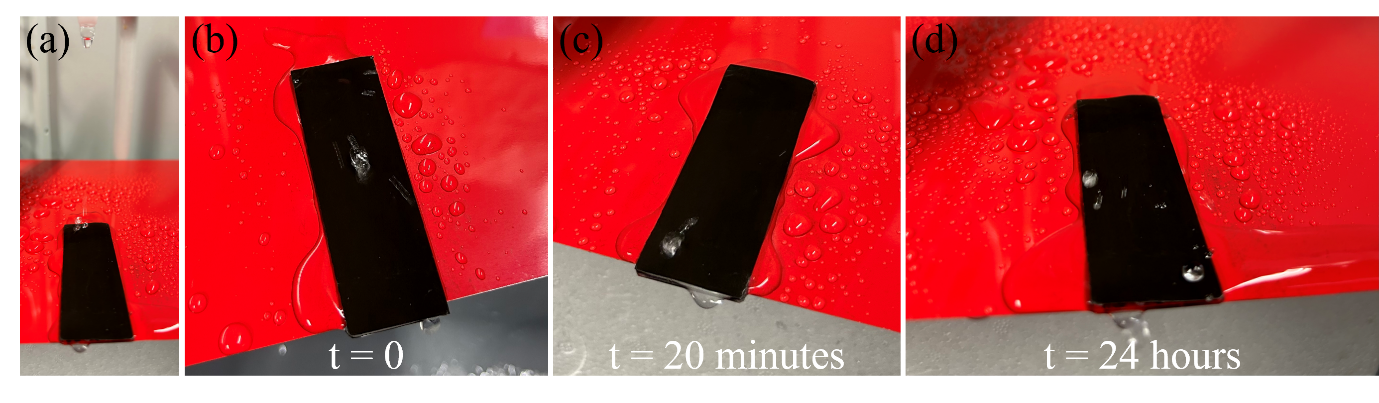
**Figure S6.** Water impact resistance test of the prepared superhydrophobic membrane. (a) Setup; Capture of water droplets rolling on the superhydrophobic membrane when (b) t=0; (c) t=20 mins; (d) t=24 hrs.


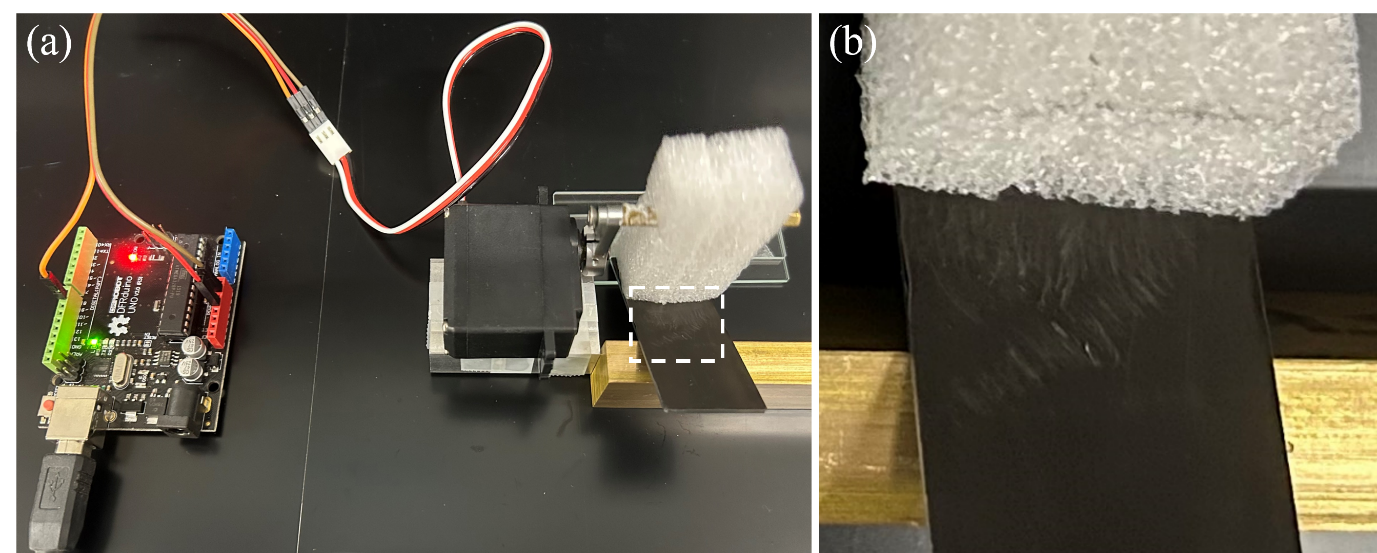
**Figure S7.** Abrasion resistance test. (a) Setup; (b) Surface appearance after abrasion.


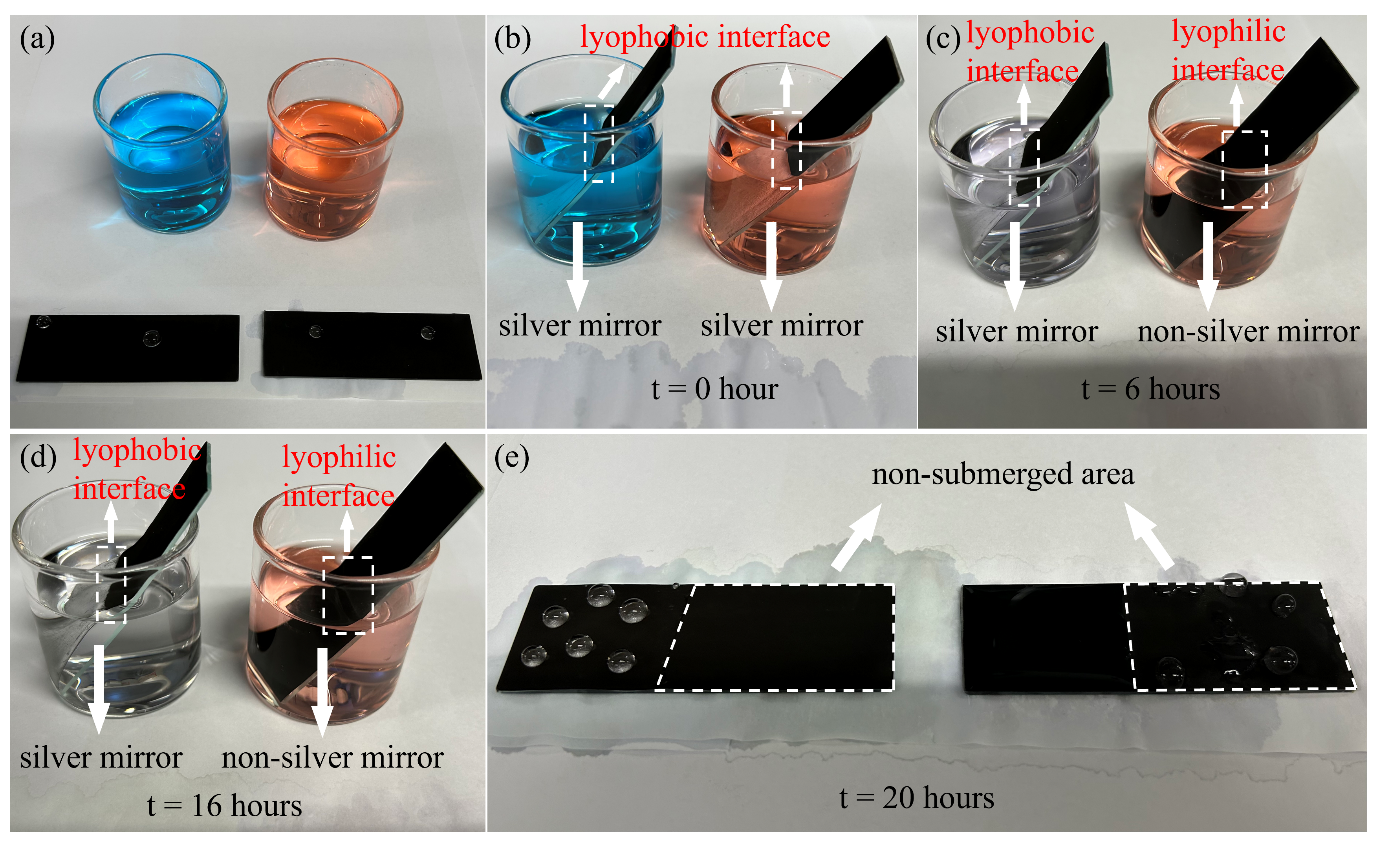
**Figure S8.** Anti-corrosive liquids capability investigation. (a) Initial states of membranes with superior liquid repellency against corrosive liquids; left: 50 mL 1 M KOH aqueous solution stained with 50 μL blue dye; right: 50 mL 1 M HCl aqueous solution stained with 50 μL red dye. (b) Membranes submerged into 1 M KOH and 1 M HCl solution; t=0 hour. Solution color and membrane wettability change over the immersion period: (c) t=6 hours; (d) t=16 hours. (e) Final states of membranes with different liquid-repellent performance; left: 1 M KOH; right: 1 M HCl; t=20 hours.


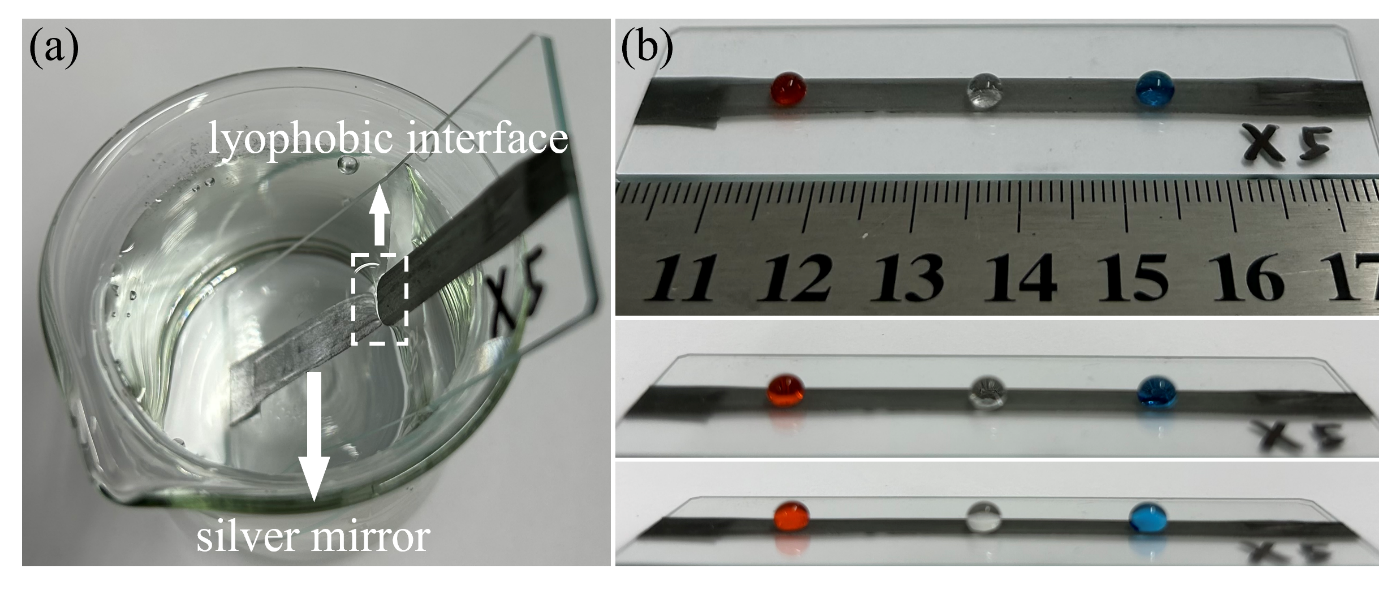
**Figure S9.** Liquid-repellent capability investigation on superhydrophobic membranes in the stretched state of 5 times in length. (a) Stretched membrane immersed in water; (b) Stretched membrane against corrosive liquids. Photos taken from different perspectives. Liquid droplets are 1 M HCl (red), water (colorless), and 1 M KOH (blue), respectively.

**Section 5.** The anticipated application of the breathable superhydrophobic membrane serving for protective shoes.


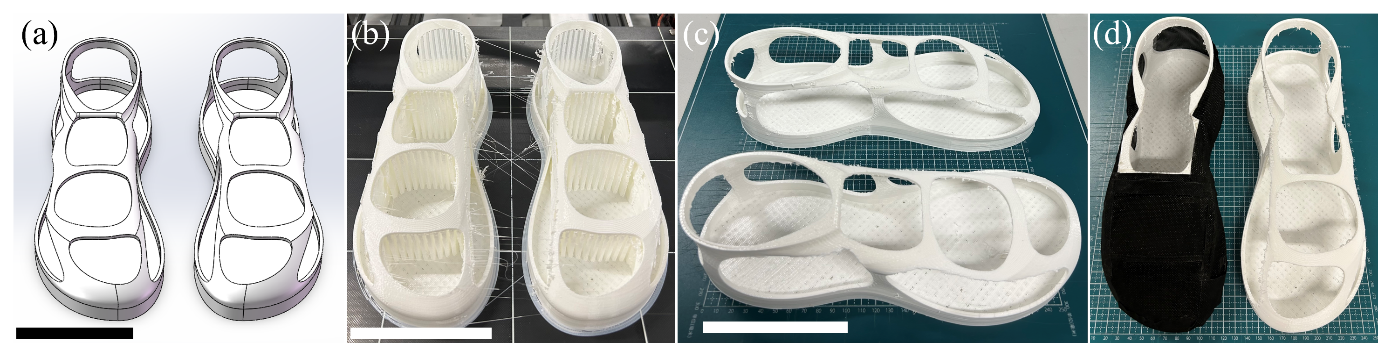
**Figure S10.** The prototype of the shoes. (a) 3D design model; (b) 3D-printed TPU shoes; (c) 3D-printed TPU shoes with the support layer removed, and (d) shoes with the prepared breathable superhydrophobic membrane pasted. Scale bars are 10 cm.

**Section 6.** Videos.

The videos are available in the attached MP4 files.
